# Supplementary material for: The Synergistic Effect of Biochar-Combined Activated Phosphate Rock Treatments in Typical Vegetables in Tropical Sandy Soil: Results from Nutrition Supply and the Immobilization of Toxic Metals
Source: Int J Environ Res Public Health. 2022 May 25;19(11):6431. doi: 10.3390/ijerph19116431 (PMC9180871; doi:10.3390/ijerph19116431)
Supplement: Supplementary file 1 [file ijerph-19-06431-s001.zip › ijerph-1685391-supplementary.pdf]

Supplementary Material

The synergistic effect of biochar combined activated phosphate rock typical vegetables in tropical sandy soil: Results from nutrition supply and immobilization of toxic metals

Zhiwei Zhang [a,b], Beibei Liu [b,d*], Zhenli He[c], Pan Pan [b,d], Lin Wu [b,d], Bigui Lin [b,d], Qinfen Li [b,d], Xinchun Zhang [b], Zhikang Wang[a]

[a] Guizhou Minzu University, Guiyang, Guizhou, China

[b] Institute of Environmental and Plant Protection, Chinese Academy of Tropical Agricultural Sciences/ Hainan Key Laboratory of Tropical Eco-Circular Agriculture/ Hainan Engineering Research Center for Non-point Source and Heavy Metal Pollution Control, Haikou, Hainan, China

[c] University of Florida, Institute of Food and Agricultural Sciences, Indian River Research and Education Center, Fort Pierce, FL, USA

[d] Danzhou Scientific Observing and Experimental Station of Agro-Environment, Ministry of Agriculture and Rural Affairs, China/National Agricultural Experimental Station for Agricultural Environment, Danzhou, Hainan, China.

[*]Corresponding author.

Email address: Beibei Liu: catas_bbl@163.com.

Additional materials and methods

Test S1. Cropping of water spinach and pepper

The experimental pots were filled with collected soil (5kg/pot). In the beginning, eighteen seeds of water spinach were sown in petridishes using filter paper drench with Millipore water. After the emergence of the radical (at least 1 mm), the seeds were transferred into an incubator and allowed to grow for 30 days under controlled condition until the plants developed 6–8 leaves. The seedlings (1 per pot) were then transferred to the pots containing collected soil following Randomized Complete Block Design (RCBD) with three replications. For the pepper, the cropping process followed the above method for further 60 days at the second cropping cycle.

Test S2. Adsorption isotherm experiment

To explore the passivation effect of various amendments on cadmium and lead in sandy soil, the adsorption capacity of sandy soil with various amendments on Cd and Pb in Millipore water was conducted through isothermal adsorption experiments. First, 0, 25, 50, 100, 500, 1000 mg/L Cd^{2+} and Pb^{2+} stock solution were prepared with 0.01 M $CaCl_2$ solution as solution electrolyte respectively. Two groups of comparative experiments were carried out. Each accurately weighed samples of 0.50 g biochar, phosphate rock, activated phosphate rock, BCPR and BCAPR amendments were placed into a 50-mL polypropylene centrifuge tube respectively (soil to solution ratio=1:50). For the other experiment group, 0.02 g biochar, phosphate rock, activated phosphate rock, BCPR and BCAPR amendments were mixed with 1.00 g sandy soil samples respectively, and then were placed into a 50-mL polypropylene centrifuge tube respectively (soil to solution ratio=1:10). Two control groups were also set up in the two groups of comparative experiments experiment respectively, and each treatment included three parallel treatments. Afterwards, 10 mL and 25 mL a series concentration level

of Cd^{2+} and Pb^{2+} solution was added to each a 50-mL polypropylene centrifuge tube in the two groups of comparative experiments respectively. The suspensions were shaken at 200 rpm for 24 h at a constant temperature of 25 °C. Finally, the Cd^{2+} concentration was determined using flame atomic absorption spectroscopy (PinAAcle 900T) after the above solution was centrifuged at 4000r/min for 10min through a 0.45μm membrane.

Table S1 Characteristics of the tested soil and amendments.

		Soil	BC	PR
	pH	5.54±0.05	9.61±0.13	9.51±0.12
Toxic metals (mg/kg)	Total As	3.91±0.12	0.62±0.06	7.90±0.46
	Total Cd	0.01±0.00	0.35±0.01	0.09±0.01
	Total Pb	9.30±0.45	5.63±0.34	4.58±0.56

BC: biochar, PR: phosphate rock.

Table S2 Adsorption parameters of Cd and Pb on single and composite amendments.

Toxic metal	Amendment	Equation<br>Ceq/q=1/kb+ Ceq/b	R^2	b<br>mg/g	k<br>L/mg	ΔG<br>kJ/mol	Sorptivity<br>L/g
Cd^{2+}	PR	y = 0.1148x + 22.962	0.9118	8.7108	0.048	7.5356	0.416
	APR	y = 0.0741x + 19.667	0.8842	13.4953	1.279	-0.6098	17.262
	BC	y = 0.0706x + 17.49	0.8817	14.1643	1.286	-0.6239	18.220
	BCPR	y = 0.0821x + 23.384	0.9022	12.1803	1.229	-0.5100	14.964
	BCAPR	y = 0.0929x + 24.634	0.9232	10.7643	1.173	-0.3960	12.630
Pb^{2+}	PR	y = 0.0214x + 0.0058	0.8312	46.7290	207.428	-13.2173	9692.877
	APR	y = 0.0234x + 0.0059	0.8094	42.7350	209.404	-13.2408	8948.885
	BC	y = 0.0291x + 0.2795	0.9888	34.3643	3.618	-3.1862	124.342
	BCPR	y = 0.0207x + 0.0521	0.9905	37.0370	19.378	-7.3439	717.702
	BCAPR	y = 0.0227x + 0.0062	0.8137	44.0529	198.218	-13.1048	8732.089

BC: biochar, PR: phosphate rock, APR: activated phosphate rock, BCPR: composite of biochar and phosphate rock, BCAPR: composite of biochar and activated phosphate rock.

Table S3 Adsorption parameters of Cd and Pb on single and composite amendments in the sandy soil.

Toxic metal	Amendment	Equation<br>Ceq/q=1/kb+ Ceq/b	R^2	b<br>mg/g	k<br>L/mg	ΔG<br>kJ/mol	Sorptivity<br>L/g
Cd^{2+}	Control	y = 0.6632x + 179.47	0.951	1.5078	0.006	-12.7341	0.009
	APR	y = 0.6356x + 129.61	0.9573	1.5733	0.008	-11.9441	0.013
	BC	y = 0.5764x + 90.674	0.9713	1.7349	0.011	-11.0949	0.020
	BCPR	y = 0.5751x + 130.13	0.9501	1.7388	0.008	-11.9353	0.014
	BCAPR	y = 0.593x + 125.75	0.9514	1.6863	0.008	-11.8539	0.014
Pb^{2+}	Control	y = 0.4214x + 1.3796	0.9999	2.3753	0.725	0.7970	1.722
	APR	y = 0.1961x + 1.0577	0.9983	5.0994	0.947	0.1348	4.829
	BC	y = 0.2221x + 2.1798	0.9933	4.5025	0.462	1.9140	2.079
	BCPR	y = 0.3888x + 1.7409	0.9991	2.5720	0.575	1.3713	1.479
	BCAPR	y = 0.3267x + 1.0912	0.9995	3.0609	0.917	0.2150	2.806

BC: biochar, PR: phosphate rock, APR: activated phosphate rock, BCPR: composite of biochar and phosphate rock, BCAPR: composite of biochar and activated phosphate rock.

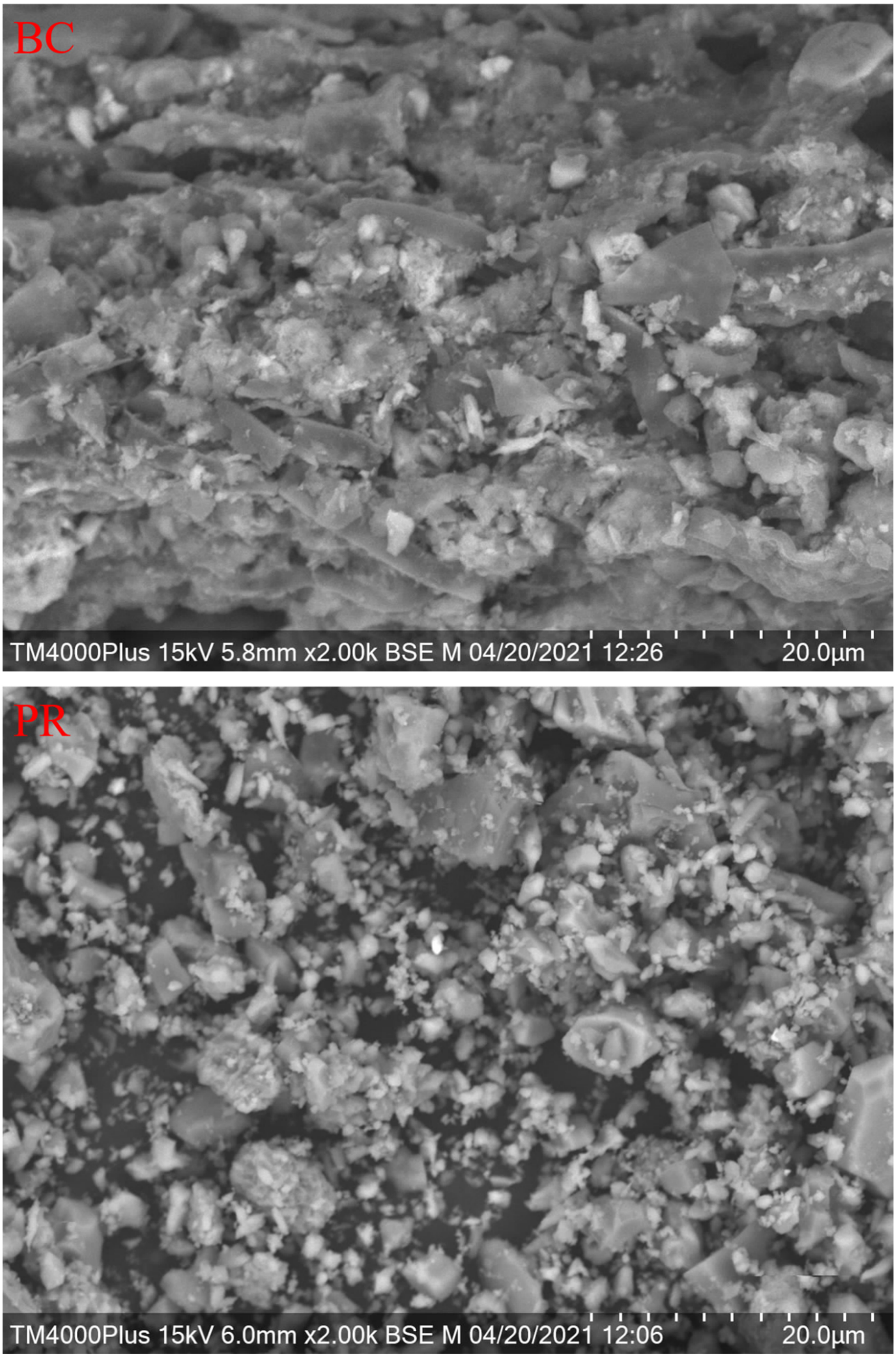
BC
TM4000Plus 15kV 5.8mm x2.00k BSE M 04/20/2021 12:26
20.0µm
PR
TM4000Plus 15kV 6.0mm x2.00k BSE M 04/20/2021 12:06
20.0µm

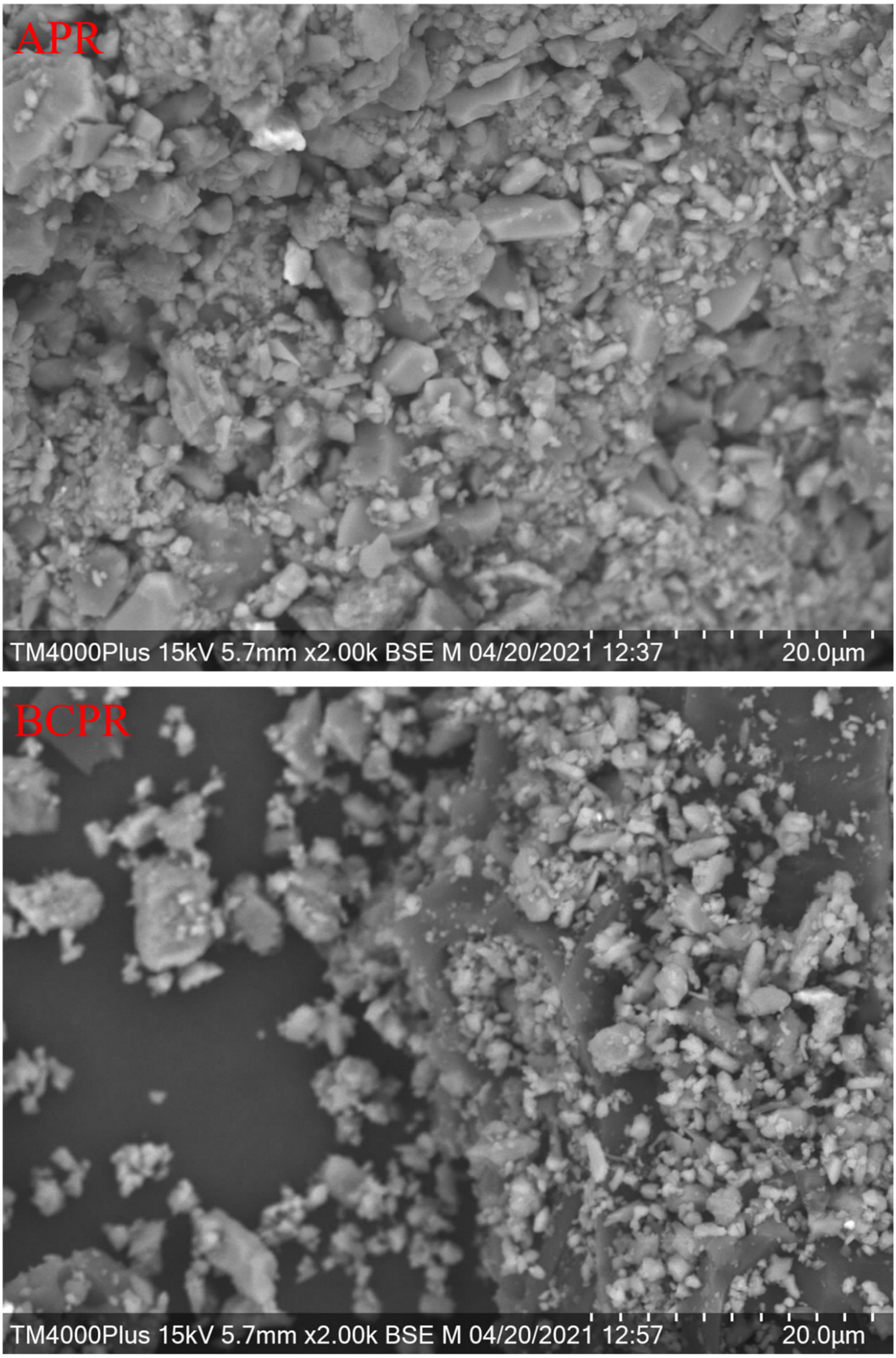

Figure S1. SEM images of different amendments in the magnification of 2.00 k. BC: biochar, PR: phosphate rock, APR: activated phosphate rock, BCPR: composite of biochar and phosphate rock, BCAPR: composite of biochar and activated phosphate rock.
